# Discursive Ethics as a Normative Foundation for Integrating Ethics into AI Clinical Decision Support Systems

**DOI:** 10.1007/s11948-026-00603-1

**Published:** 2026-05-14

**Authors:** José-Félix Lozano

**Affiliations:** Instituto Ingenio (CSIC - UPV), Universitat Politècnica de València, Camino de Vera S/N, Valencia, 46022 Spain

**Keywords:** AI ethics, Discursive ethics, Clinical decision support systems, Human agency, Communicative rationality

## Abstract

The rapid integration of Artificial Intelligence (AI) in critical sectors such as healthcare has raised significant concerns regarding human autonomy and the risk of delegating vital decisions to opaque artificial systems. This paper examines the normative foundations required for the responsible integration of ethical principles into AI-based Clinical Decision Support Systems. First, it reviews key descriptive models of human moral judgment—rationalist, intuitionist, and integrative approaches—and highlights their relevance for understanding how humans make ethical decisions under conditions of uncertainty. Particular attention is given to the Actor–Deed–Consequences (ADC) model, which captures the plural and intuitive structure of moral evaluation and offers a promising basis for informing ethical guidance functions in AI systems. Building on this analysis, the paper argues that descriptive models alone are insufficient to secure the legitimacy of AI-assisted clinical decisions. To address this gap, it proposes a discursive ethics framework grounded in the work of Karl-Otto Apel, Jürgen Habermas, and Adela Cortina. This framework emphasizes communicative rationality, stakeholder involvement, transparency, and accountability as essential conditions for ethically acceptable AI deployment. The paper concludes that AI-based CDSS should be developed as dialogical and process-oriented tools that support human judgment. Embedding discursive ethical procedures within AI architectures provides a robust normative pathway for ensuring that technological innovation in healthcare enhances human agency, safeguards dignity, and strengthens trust in clinical practice. Keywords: AI Ethics, Discursive Ethics, Clinical Decision Support Systems, Human Agency, AI Act, Communicative Rationality.

## Introduction

The ethical reflection on science and technological advances has a long tradition and continues to raise questions we have yet to answer. When Hans Jonas stated in the late 1970s, at the height of the nuclear race, that “The promise of paradise that science once offered has become a threat” (Jonas, 1979), the German philosopher K. O. Apel added that this required us, with even greater urgency, to develop an ethical framework equal to these challenges (Apel, 1973, Apel, 1988, Apel, 1997). In our view, both statements remain valid. Power implies responsibility, and the power of Artificial Intelligence (AI) to transform life demands ethical reflection commensurate with that power.

Today, the application and use of Key Enabling Technologies (KET), advances in communication technology, and the synergistic convergence of the Internet of Things (IoT), Big Data, and Artificial Intelligence (AI), which have enabled digital hyperconnectivity and the collection, processing, and use of massive data, are providing significant advantages in fields such as medicine, communication, education, industry, and governance. Specifically, the benefits of AI are well known and have a significant impact on the quality of life of individuals and society (Commission, 2018; Floridi et al., 2018; OECD, 2019; Veale et al., 2018).

Since the discipline’s inception in 1956, advances in AI have continued to surprise us. Although expectations have generally outpaced reality (López de Mántaras, 2017), it is possible, as stated in the report by the High-Level Expert Group on AI Ethical Advisory Board EU Commission, that “Artificial Intelligence (AI) is one of the most transformative forces of our time and is set to reshape the social fabric” (Commission, 2018). Following Mustafa Suleyman’s words, this “new wave” of technology is bringing humanity to a pivotal change that will affect all areas of life, and it is unstoppable, though governable (Suleyman, 2023).

It is hard to dispute some of the advantages and possibilities offered by AI, such as greater resource optimization, expanded spaces for participation, and increased analytical, diagnostic, and predictive capabilities across various fields, among many others. However, ongoing scandals over the illicit use of private data (Véliz, 2020); homophobic, xenophobic, misogynistic, or poverty-based biases and the behavioural relativism of algorithms; the depersonalization and diffusion of responsibility for the actions and decisions of institutions, organizations, and companies; the commercialization and commodification of human beings; the rise in diseases linked to the excessive control underlying hyperconnectivity processes (stress, anxiety, depression, low self-esteem, karoshi, etc.); and the human obsolescence underlying technological imperialism all make it urgent and necessary to discuss the risks, limits, and consequences of AI (Nowotny, 2021).

To these risks, we should add the severe threat to credibility and trust posed by the development of generative AI. Fake news, as well as counterfeit images and video montages, are affecting individuals (including minors and vulnerable people in some cases) and creating confusion and distrust among citizens. In addition to these individual negative effects, we must also consider the medium- and long-term social impact that the development of this technology may have. Its impact on inequality, freedom, and democracy, among others, is no minor issue (Coeckelbergh, 2023).

Framing these questions more substantively and practically requires to address four fundamental points in the understanding how AI affects human dignity and flourishing: “who we can become (autonomous self-realization); what we can do (human agency); what we can achieve (individual and societal capabilities); and how we can interact with each other and the world (societal cohesion)” (Floridi et al., 2018. p. 690).

These four issues focus on a general question: to what extent does AI assist us in decision-making, or does it substitute us in it? Spanish philosopher Adela Cortina argues that the ethical question of AI must begin by recognizing (and attempting to articulate) “the vast difference between using intelligent systems (whether machines, algorithms, robots) to aid in decision-making and delegating to those intelligent systems meaningful decisions that affect human lives and nature” (Cortina, 2019, p. 381). The fact is that individuals increasingly outsource more of their decisions to AI decision support systems, even those that carry vital human dimensions. This is a decisive point: the essence of ethics is freedom, and any social, political, or technological initiative that undermines human freedom runs counter to human development and justice.

In this article, we will reflect on the ethical challenges of Clinical Decision Support Systems and propose a process for integrating ethical principles into them. The process will be based on the discourse theory developed by German philosophers K. O. Apel (Apel, 1973, Apel, 1988, Apel, 1997) and Jürgen Habermas (Habermas, 1968, 1981, 1983, 1992, 2021), and the Spanish philosopher Adela Cortina (Cortina, 1986, 1990, 1996, 2010).

## How Do People Really Make Moral Decisions?

Before proposing ways to develop artificial systems that can make or support decisions that incorporate the moral dimension, we must first understand how the human cognitive system operates in moral decision-making. Understanding how people make moral decisions is, therefore, a necessary first step. In this section, we present the three most influential models of ethical decision-making developed over the past few decades: rationalist, intuitive, and heuristic.

We live because we make decisions, and live in a certain way because we make decisions based on certain ideas. The concern with reasons for action and with “correct” criteria for decision-making has a long history in philosophy. The problem is identifying the criteria for a correct decision and determining whether we can establish a hierarchy among them. Practical reason aims to think to act; just as speculative reason seeks methods to understand, practical reason seeks grounds to determine the will. The fact that humans have the capacity to determine their causality through the representation of rules implies an acceptance of the awareness of freedom of will as a factum (Kant, 1785, 1787). This capacity to act according to rules is the key to practical reason (Habermas, 1981, 1992).

We can encounter several definitions of ethical decision-making. “Ethical decision is defined as a decision that is both legal and morally acceptable to the larger community” (Jones, 1991). This general definition leaves several questions open. Carlson presents a more precise version and defines ethical decision-making as “a process by which individuals use their moral base to determine whether a certain issue is right or wrong” (Carlson et al., 2002, p. 536). This definition includes three dimensions: a) the process to reach a decision, b) the agent’s intention and c) the consequences. All three are key elements for understanding how people make decisions and for assessing the ethical level of a concrete decision.

In recent decades, the findings of neuroethical research appear to open new pathways in understanding practical rationality (Clarke, 2013; Evers et al., 2017; Illes, 2017; Levy, 2007; Safire, 2007). These new pathways, which revalue the emotional and intuitive dimensions of cognitive ability, are highly relevant for decision-making in business and professional settings.

### “Rationalist” Models of Ethical Decision-Making

Even within the most mathematical approaches to decision-making, such as Analytic Hierarchy Process (AHP) (Saaty, 1987) or Multi-Criteria Decision Analysis (MCDA), it is recognized that making a decision is not simply a matter of selecting the best alternative but involves complexity and multidimensionality that requires a high level of intellectual effort, reflection, and abstraction, with mindsets and emotions playing a central role.

In moral philosophy, the dominant model until two decades ago was the rationalist model, in which deterministic, causal, and functional explanations were overvalued. The American moral philosopher James Rest proposed a model of individual ethical decision-making and behaviour comprising four steps: moral awareness, moral reasoning, moral intent, and moral behaviour (Rest, 1986). This model follows a four-phase causal linear process: 1) recognition of moral issues, 2) moral judgment, 3) moral intention, and 4) moral conduct.

This approach is based on Kohlberg (1981) and Rest’s (Rest & Narvaez, 1994) theory of moral judgment evolution. It focuses on analyzing how organizational context factors and the moral intensity of issues influence all phases of decision-making (Fallon & Butterfield, 2005). For Jones (1991), the key to ethical decision-making lies in perceiving the intensity (severity) of the ethical issue at hand. From the same assumptions of linear-causal rationality, Trevino’s model suggests that ethical decision-making results from the interaction between agents’ moral judgment and situational factors (Trevino, 1986).

These models assume that moral judgment and moral reasoning function analytically and sequentially, and that the key to ethical behaviour lies in the “external” factors that influence moral judgment.

### “Intuitionist” Models of Ethical Decision-Making

In the last few decades, the rationalistic model has been questioned by advances in cognitive neuroscience research (Damasio, 1994). Conclusions from neuroscience and cognitive psychology research confirm that our cognitive system is less rational than we tend to think, and that intuition plays a pivotal role in human decision-making (Gigerenzer, 2008; Kahneman, 2011).

There are four significant limitations identified in the rationalistic approach: 1) Uncertainty and ambiguity. Ethical dilemmas often present simultaneous different interpretations and a severe lack of information and precision. We tend to have a positive illusion about the consequences of our decisions and underestimate negative impacts. We are also imprecise in defining the ethical issue, and there is significant variation in how situations are defined as ethical or unethical. 2) Deliberation and rationalization. The rationalist model suggests that deliberation and rationalization are prerequisites for moral awareness, and that the behavioural response should thus align with the moral standpoint. However, recent advances in cognitive psychology demonstrate that individuals engage in much less pre-deliberation and rationalization than we tend to believe. 3) Objectivity of situations. Rationalist approaches suggest that the process starts with a problem and that subjects react to pre-existing ethical dilemmas. However, research shows that individuals develop subjective interpretations of issues beyond objective characteristics. 4) Moral reasoning and judgment. Critics argue that moral judgment does not result from moral reasoning but rather that we respond intuitively to moral issues and then justify them (Haidt, 2001).

The intuitionist approach aims to overcome these limitations. Moral intuition can be defined as “the sudden appearance in consciousness of a moral judgment, including an affective valence (good-bad, like-dislike), without any conscious awareness of having gone through steps of searching, weighing evidence, or inferring a conclusion” (Haidt, 2001, p. 818)

Based on advances in cognitive psychology and neuroscience, two interesting models challenge and aim to surpass rationalist models: the “Sensemaking-Intuition Model” (SIM) presented by Sonenshein (2007) and the Sensemaking model by Thiel et al. (2012).

Sonenshein proposes his Sensemaking-Intuition Model (SIM), which is composed of three levels: issue construction, intuitive judgment, and explanation and justification (Sonenshein, 2007). This model posits that individuals construct issues from social stimuli in uncertain, ambiguous environments, heavily influenced by their expectations and motivations. The key is how individuals make sense of contextual stimuli by creating stories. People create stories to make sense of contextual events, regardless of their accuracy, and think more with metaphors than with data. Along these lines, Gigerenzer’s “ecological rationality” suggests that ignoring some information is the best decision-making strategy in many areas and that humans use heuristics quite successfully. He defines the rationality of heuristics not by optimization and content-blind norms, but by the degree to which they are adapted to the environments (Gigerenzer, 2008).

The second step is intuitive judgment. Research has shown that individuals rarely change their initial response, even when presented with new evidence. Neurological studies suggest that discursive cognitive processes are mainly used to rationalize intuitions rather than make active judgments. The Sensemaking-Intuition Model assumes that judgment is instantaneous and that the basis of moral judgment lies in the subject’s affective reaction to the issue. Once a situation is categorized as morally relevant, we automatically respond with affective reactions, shaped by individual experiences and social pressures. This moment engages the reflective cognitive system.

Finally, the third step is explanation and justification. Individuals justify and rationalize their intuitions after the fact, using rational analysis tools. We describe our decisions in rationalist terms, striving for coherence in our narrative (our decision or action) rather than faithfulness to the facts. As Kahneman states, “[…] when people believe a conclusion to be true, they are more likely to believe arguments that seem to support it, even if those arguments are unreliable” (Kahneman, 2011). Coherence matters more than reliability or information accuracy.

Thiel et al.‘s model argues that the traditional approach is inadequate for understanding how professionals make decisions in conditions of uncertainty and ambiguity, and that sensemaking is a more suitable model. Sensemaking is a cognitive process by which mental models are created that overcome information deficits and imprecise assessments, contributing to more effective evaluations. They propose that fostering responsible decision-making involves developing cognitive sensemaking strategies, including emotional regulation, self-reflection, forecasting, and information integration.

In conclusion, decision-making models inspired by rational choice theories are an inadequate framework for understanding how people respond to ethical dilemmas in conditions of uncertainty and confusion. Sensemaking models are better equipped to deal with real ethical challenges.

### The ADC Model of Moral Judgment and Decision-Making

The third model of moral judgement and decision-making we want to present is the one proposed by Racine and Dubljević (Dubljevic et al., 2018; Dubljević & Racine, 2014; Racine et al., 2017). The Actor-Deed-Consequences (ADC) model of moral judgment is an integrative approach that, in many respects, surpasses the two approaches presented.

The ADC-model predicts that moral judgment consists of concurrent evaluations of three intuitive components: the character of a person (Agent-component, A), their actions (Deed-component, D), and the consequences in the situation (Consequences-component, C). It means that when we make a moral judgement, we rely upon three types of intuitions. Indeed, we look at three directions: a) One is the attention to the agent. We assess their intention, benevolence, human quality and character, b) Another is the evaluation of the action in itself. We quickly evaluated an action based on shared social conventions acquired during our socialization process, and c) finally, we try to estimate the possible consequences of the action/decision. A case in point is that we make different moral evaluations of these scenarios:

A benevolent person (A+) lies (D-), and the consequence is that an innocent person gets injured (C-).

A delinquent (A-) tells the truth (D+); consequently, another delinquent gets free (C-).

An honourable person (A+) keeps their promise (D+), resulting in a company going bankrupt (C-).

Dubljević and colleagues said that understanding moral judgment in this way allows us to overcome the dualistic approach of intention versus consequence. This ADC model integrates the virtue approach (moral assessment of the actor), the deontological approach (moral assessment of the action independent of consequences), and the utilitarian approach (moral assessment of the action based solely on consequences).

Contrary to traditional disputes about single-component theories in moral philosophy and to some extent in moral psychology, the results of the empirical research developed by Dubljević and colleagues suggest that intuitions underlying precepts from all three dominant ethical theories (virtue ethics, deontology, and consequentialism/utilitarianism) are operative (in terms of a stable and significant contribution of each component of moral judgment) (Dubljevic et al., 2018).

After presenting these three main approaches to moral judgment, there are two caveats before reflecting on the integration of ethics in AI Decision Support Systems. First, the three moral judgment models presented here are descriptive and not normative. It means they try to understand and describe how people judge right and wrong, good or bad. However, they do not propose how people should reflect on moral issues. Second, understanding how people make moral judgments is a necessary first step for a responsible integration of ethics in AI. Models of human judgment and decision-making are crucial for informing the ethical guidance functions in AI.

## AI in Health Care: A Challenging Context

As Floridi and his collaborators warned some years ago (Floridi et al., 2018), AI can be used to promote human self-fulfilment, enhance human capabilities, increase social capacities, and cultivate social cohesion. At the same time, however, it can be misused to devalue human capabilities, eliminate human responsibility, reduce human control, and erode human self-determination.

AI systems are making decisions in critical areas like healthcare, justice, and warfare, where they face ethical dilemmas. There is a substantial debate about the role of AI in ethical decision-making in fields such as health, justice, and social welfare. While significant advantages are acknowledged, such as the ability to integrate and process more information faster than humans and the use of objective criteria, there are also serious risks.

In recent years, health care systems have been under increasing pressure, including limited funds and insufficient resources for medical care (Awad et al., 2018; Sauerbrei et al., 2023). In this specific context, AI applications (robots, Clinical Decision Support Systems, etc.) play a crucial role. These systems assist physicians in medical diagnostics, patient monitoring, and learning healthcare systems. AI-applications are currently utilized for three main objectives: a) providing diagnoses and predicting treatment outcomes based on relevant clinical data; b) researching patient populations to assess the risks of non-compliance with prescribed management plans for individual patients; and c) generating knowledge bases to enhance system-wide efficiencies and improve patient outcomes in learning healthcare systems (Lysaght et al., 2019, 2024). As Mishra and colleagues acknowledged, “The potential benefit of such systems is vast—they can lead to better clinical and health outcomes not just because they perform at a superhuman level for some tasks, but also because they may facilitate greater access to health care in populations and low-resource settings in which previously access to health care was limited” (Mishra et al., 2021).

In clinical decision-making, AI has demonstrated exceptional capabilities in predicting and classifying diagnoses while delivering actionable recommendations and insights (Khosravi et al., 2024). Early detection of pathology has become a key tool for optimizing processes in healthcare settings, enabling efficient treatment adjustments and improving survival rates in some patients (Davoodi & Moradi, 2018). This is why machine learning algorithms are increasingly playing an important role in decision-making in healthcare and biomedical engineering (Rubinger et al., 2023). With the advancement of technology, several statistical analysis methods and machine learning techniques have emerged, proving effective in improving the accuracy of clinical outcome prediction, optimising decision-making, and offering more personalised approaches tailored to individual patient needs.

Although the advantages of AI systems and robotics in clinical care and medical research are undeniable, several ethical concerns and risks are emerging:

Dehumanization and depersonalization of the patient-doctor relationship. The increasing reliance on technical systems and data can distance healthcare professionals from the human experience of illness. There is also a high risk of losing empathy and compassion (Kerasidou, 2020; Sauerbrei et al., 2023). Reliance on AI models has been argued to be in tension with the ideals of patient-centered care in two main ways: a) they are not sensitive to patient values and have difficulties to comprehend social, psychological, and spiritual dimensions, and b) they are opaque and function like a black box where transparency and explainability is highly complicate and difficult to understand by clinicians (Mishra et al., 2021).

Erosion of patient autonomy and informed consent, especially when AI decisions are opaque or when patients are unaware of how their data is used in automated processes. The concern is that patients may lose their ability to make decisions when systems that make decisions do not take their interests, expectations, or values into account. This, coupled with technical paternalism, in which a technical system makes correct decisions based on thousands of complex data points, can result in patients having less effective decision-making capacity about their health and foster paternalism, as healthcare professionals are likely to defer decisions to AI tools.

Reinforcement of biases and inequalities represents a central ethical challenge in healthcare AI. While machine learning algorithms are often promoted for their efficiency and accuracy, AI systems trained on biased or incomplete datasets risk perpetuating or amplifying existing social and structural disparities in healthcare access and outcomes (Ferrer et al., 2020; Giovanola & Tiribelli, 2023; Goirand et al., 2021). In healthcare applications, AI systems must be designed to mitigate biases in data and prevent model inaccuracies that could lead to unfavourable treatment of individuals based on characteristics such as economic status, race, gender, disability status, sexual orientation, or political affiliation (Mennella et al., 2024).

The dominance of a utilitarian and cost-efficiency mindset, in which economic or algorithmic efficiency may take precedence over patients’ rights, dignity, and individualized care, has significant impacts at both the macrosocial and individual levels. At the macrosocial level, there is concern that the use of AI in healthcare could widen health inequalities by allocating resources to those who can afford them rather than to those who need them. At the individual level, this mindset could lead to people being treated based on their financial capacity rather than their medical needs. While there is some optimism that AI could open new avenues for delivering care globally, making services more efficient and effective, particularly in low- and middle-income countries (LMICs) (Kerasidou, 2021), the experiences of the past decades and the trend towards the commodification of healthcare make us sceptical about the future.

The erosion of trust in health professionals is a growing concern, as clinical decisions are increasingly mediated—or perceived to be determined—by machines rather than human judgment. Even if AI systems already surpass human performance in certain clinical tasks, therapeutic trust in health professionals must rest not just on their epistemic authority but also on their demonstrated commitment to care and their ability to offer a holistic, individualized approach to each patient (Dlugatch et al., 2024; Kerasidou, 2020). Additionally, the use of ‘black-box’ systems could diminish physicians’ comprehension of medical processes, challenge their epistemic authority, and ultimately erode patient trust (Maris et al., 2024). If public trust in professionals shifts toward blind confidence in AI systems, it paves the way for paternalism and technological determinism in the relationship between patients and healthcare providers.

The diffusion of responsibility in AI-assisted clinical decisions creates ethical and legal ambiguity, particularly when accountability for errors or adverse outcomes becomes unclear. Moral responsibility requires two essential capacities: a moral capacity—the intention or willingness to act—and an epistemic capacity—an understanding of the potential consequences. Since AI systems are not moral agents, only human healthcare professionals can be held truly accountable (Cervantes et al., 2020; Floridi & Sanders, 2004; Graff, 2024; Nyholm, 2018; Véliz, 2021). However, when health professionals rely heavily on outputs from systems that are impossible to trace back or understand (such as neural networks), the extent to which responsibility can be attributed to them becomes significantly constrained.

## AI Decision Support Systems: The Ethical Challenge

Let us not lose sight of the goal of our article. What we seek here is the best way to effectively and legitimately integrate ethical principles into decision-making for AI applications in the clinical context.

To achieve this goal, we must first understand the mechanisms of human decision-making and the technical functions of these systems, while remaining mindful of their bidirectional interaction. The way we process information to make decisions inherently influences the design of artificial support systems; conversely, Intelligent Decision-Support Systems (IDSSs) actively shape human ethical decision-making (Poszler et al., 2024).

### Intelligent Decision Support Systems

Decision Support Systems (DSS) encompass a diverse set of computational frameworks designed to assist decision-makers in synthesising data, models, and specialised knowledge to address semi-structured, ill-structured, or unstructured problems.

Intelligent Decision Support Systems (IDSS) are increasingly deployed in high-stakes domains, including health (Clinical Decision-Support Systems), justice (sentences and parole decision systems), military (autonomous weapon systems), social services (Compass System), and artificial moral advisors (Delphi bot), where the intersection of technical efficiency and ethical deliberation is essential. Undoubtedly, these systems have an enormous capacity to manage information quickly and objectively. Such systems may allow individuals to make more informed, value-aligned, and rational decisions by providing them with information on morally relevant factors in a decision situation. The identified advantages of IDSS include: autonomy (the ability of systems to make decisions without human instructions or interference), efficiency (the technological superiority of systems in accessing, integrating, and analyzing complex data in an aggregated manner), reliability (systems often provide consistent responses to similar inputs), value neutrality (systems are impartial and dispassionate, unaffected by stress, fear, or biases), and flexibility (systems can learn from past decisions).

Indeed, some authors consider that AI systems could enhance our moral capacity (Lara & Deckers, 2020). Although this is considered too optimistic, we can identify among the expected advantages of using these systems improvements in deliberation, motivation, autonomy, and moral action. However, the negative medium- and long-term effects could include moral deskilling, restricted moral progress, and a moral responsibility gap. Essentially, the major risk is falling into the automation bias, the tendency to accept, by default, the judgments of automated systems as final and superior to those of humans (Poszler et al., 2024).

There is intense controversy over whether artificial moral agents should be developed/utilized for ethical decision-making at all (Poszler et al., 2024). However, the fact is that this technology is already in use across a variety of critical sectors, often embedded within algorithms that quietly navigate high-stakes trade-offs. From autonomous vehicles programmed to make split-second “collision choices” to triage software in healthcare and automated risk assessment tools in the legal system, AI is no longer a theoretical ethical subject; it is a functional participant in the moral landscape. Consequently, the conversation is shifting from whether these agents should exist to how we can embed ethical criteria in the AI Decision Support Systems.

As Tasioulas explains, integrating ethical principles into AI can be approached from two perspectives: bottom-up or top-down. Top-down (or deterministic or closed-rule) algorithms control an AI system or robot’s behaviour by means of a predetermined program; whereas Bottom-up (or stochastic) algorithms, by contrast, enable a robot to ‘learn’ from experience and revise its algorithm over time (Tasioulas, 2019).

Specifically, regarding IDSS, Franziska Poszler and Benjamin Lange recently conducted a systematic literature review to answer the following questions: 1) In what way can intelligent decision-support systems hinder or facilitate humans’ ethical decision-making? 2) By what mechanisms do intelligent decision support systems hinder or facilitate humans’ ethical decision-making? (Poszler et al., 2024).

IDSS can adopt either a process-oriented navigation mode or an outcome-oriented process. “While process-oriented navigation is characterized by neutral informational, analytical and suggestive features, the features of outcome-oriented navigation are rather directive and value-laden as specified by the IDSS’s user (i.e., participatory) and/or programmer (i.e., heteronomous). As a result, process-oriented navigation aims to guide users through their ethical decision-making process, while outcome-oriented navigation aims to direct users toward a predetermined ethical decision outcome.” (Poszler et al., 2024, p. 13). ‘Process-oriented navigation’ and ‘outcome-oriented navigation’ have highly relevant implications for ethical decision-making and the role humans are assigned in it. While process-oriented programming approaches “invite” individuals to reflect on the process and the outcomes, heteronomous outcome-oriented programming fosters moral unreflectiveness. This occurs because it assumes that the programmed goal is valuable and that humans are incapable of understanding (and improving) the machine’s reasoning/deliberation process.

Poszler and Lange concluded that IDSSs that strive for outcome-oriented navigation do not help users learn to reason (ethically) in this way but force users to refrain from extensive ethical deliberation, and that IDSSs’ contribution to humans’ autonomy enhancement decreases as they move from process-oriented to outcome-oriented navigation. This conclusion has practical relevance as it provides technology companies with information on what are (un)intended sources of influence within IDSSs (i.e., the system’s human creators and the system itself), what are different operations and features that can be adopted in the design of systems (i.e., process-oriented navigation and outcome-oriented) and what are corresponding ramifications for the ethical decision-making on an individual and societal level.

### Clinical Decision Support Systems

Clinical decision-making is a particularly sensitive area due to its inherent complexity. The principle of autonomy in bioethics requires patients to be informed and empowered to decide how they wish to be treated. However, patients often face significant challenges, such as pain, fear, intense emotional stress, and cognitive limitations, which can hinder their ability to make informed decisions. Family members who might assist or assume decision-making responsibilities can also experience considerable pressure, potentially compromising their ability to respect patients’ preferences. Meanwhile, doctors must navigate the dual challenge of processing vast amounts of complex medical information while respecting patients’ and/or their families’ decisions (Benzinger et al., 2024). In this context, Clinical Decision Support Systems (CDSS) play a crucial and invaluable role.

“The purpose of CDSS is to support physicians and patients by providing a large amount of clinical-diagnostic information for the joint decision-making process, which is selected individually and case-oriented by integrated software systems” (Ethikkommission, 2021, p. 1). In clinical and health decision-making, AI has shown exceptional capabilities in predicting and classifying diagnoses, as well as delivering actionable recommendations and insights (Khosravi et al., 2024).

AI-assisted CDSS is currently used for three main objectives: a) to provide diagnoses to patients and predict treatment outcomes based on relevant clinical data; b) to research patient populations to calculate the risks of non-compliance to prescribed management plans for individual patients; and c) to generate knowledge bases to improve system-wide efficiencies and patient outcomes in learning healthcare systems (Lysaght et al., 2019). However, it also represents some challenges. As AI algorithms develop and become more ubiquitous, there are concerns that doctors will become over-reliant on AI assistance and complacent in their decision-making. Linked to this, another major concern about AI-assisted CDSS is how the machines reach decisions, and which decision should prevail when there is disagreement between the CDSS and the medical profession. The lack of transparency (referred to as the “black box”) is a new phenomenon with severe implications for health, including legal liability. The fact that the functionality and the achievement of a result are, in principle, no longer comprehensible even to developers is a new phenomenon in technical systems. These tools must be designed to be explainable so that doctors can understand how the machine arrived at the recommended diagnosis (e.g., the use of an X-ray machine) (Lysaght et al., 2019).

One of the central ethical issues concerns the impartiality and fairness in clinical decision-making (Giovanola & Tiribelli, 2023). Fairness requires much more than just non-discrimination. It is primarily understood as the absence of biases and requires removing four specific types of bias: model design, training data, interactions with clinicians, and interactions with patients (Ferrer et al., 2020).

Ultimately, the integration of AI into clinical environments must be viewed not as a replacement for human judgment, but as a reflective help. While CDSS offers unprecedented efficiency and data-driven insights, the risks of automation bias and ‘black box’ opacity necessitate a steadfast commitment to process-oriented design. Only through a reflective and dialogical integration can we ensure that these systems empower clinicians and patients alike, safeguarding the essential human dignity.

## Integrating Ethical Principles in AI Clinical Decision Support Systems

The integration of ethical principles into clinical decision-making systems is a task as formidable as urgent. Historically, the transition from theoretical reflection to practical application has challenged ethical theory; however, this shift is particularly arduous in the field of AI for several reasons. First, the field is characterised by unprecedented novelty and rapid evolution. Second, a significant epistemological divide exists between technical and humanistic disciplines. Third, there is a fundamental axiological tension between core ethical values—such as autonomy, privacy, equity, safety—and prevailing economic imperatives, including profitability and efficiency.

Experts generally agree that transparency, justice, non-maleficence, responsibility, and privacy form the bedrock of responsible AI (Hohma & Lütge, 2023). Beyond these core values, nine specific principles are widely recognized as essential: (1) fairness and non-discrimination, (2) privacy, (3) safety and security, (4) human control of technology, (5) transparency and explainability, (6) accountability, (7) the promotion of human values, (8) professional responsibility, and (9) sustainable development. These principles are categorised into three functional dimensions: mitigating undesirable outcomes, ensuring responsible conduct, and advancing human and environmental well-being. However, a significant challenge lies in effectively integrating these values into decision-support systems across specific contexts.

Despite these difficulties, significant scholarly contributions have sought to bridge this gap by embedding traditional ethical frameworks, such as virtue ethics, deontology, and consequentialism, into algorithmic architectures (Geisslinger et al., 2023; Poszler et al., 2023, 2024; Wernaart, 2021).

Goirand and colleagues conducted an extensive literature review of how ethics frameworks have been implemented in AI-based Health Care Applications (AIHA), how these implementations have been evaluated, and whether they have been successful (Goirand et al., 2021). They concluded that there is no one-size-fits-all approach and that contextualization is needed.

Here we would like to present two interesting processes for ethical integration in AI-assisted CDSS: a) procedural and substantive values integration presented by Lysaght and colleagues, and b) ADC integration proposal developed by Dubljević, Racine and colleagues.

### Lysaght Integration Proposal

Lysaght and colleagues adopt a predominantly normative approach and argue that the ethical development and implementation of AI-assisted CDSS in healthcare should be guided by two broad categories of values: substantive values and procedural values (Lysaght et al., 2019).

Substantive values refer to the core ethical principles that ought to be embedded in the design and use of CDSS. The essential substantive values are professional integrity, justice, and public benefit.

Professional integrity requires that clinicians’ qualifications, responsibilities, and professional judgment remain central to clinical decision-making. AI-assisted CDSS should be designed to support and enhance clinicians’ cognitive capacities rather than replace or override their expertise. In this sense, such systems must respect the primacy of human judgment and ensure that clinicians retain meaningful control over decisions affecting patient care.

Justice, understood primarily as fairness and equity, demands careful attention to the risk that AI systems will reproduce or amplify existing social biases. Given that AI-assisted CDSS often rely on historical data, there is a significant danger that biased algorithms may disproportionately harm disadvantaged populations. As Lysaght and collaborators emphasise, developers should be transparent about these limitations and incorporate mechanisms to detect emergent biases, enabling timely feedback and system improvement.

Public benefit highlights the need to consider health-related decisions not only from an individual perspective but also in terms of their broader societal impact. Public and private interests in healthcare are deeply interconnected, particularly in publicly funded health systems. While respect for individual rights remains fundamental, there may be exceptional circumstances—such as public health emergencies exemplified by the COVID-19 pandemic—in which the collective good justifiably takes precedence over individual preferences.

In addition to substantive values, Lysaght et al. identify procedural values, which concern the processes through which AI-assisted CDSS are developed, implemented, and governed: Transparency and Accountability.

Transparency requires that AI algorithms be explainable to the greatest extent possible, allowing clinicians and relevant stakeholders to understand how recommendations are generated and to assess their appropriateness in specific clinical contexts.

Accountability entails that CDSS applications are traceable and auditable, with clear lines of responsibility. Crucially, the ultimate responsibility for clinical decisions must remain with the healthcare professional, ensuring that the use of AI does not obscure or dilute professional accountability.

According to Lysaght and colleagues, the concrete integration of these principles requires developing a discursive balancing approach that follows four steps.

First step: Stating the problem or ethical issues

The first issue is to adequately identify the ethical problem. To do so, it is essential to be clear about the overarching objectives of the clinical profession: a) respecting patient autonomy; b) ensuring that patients are not subjected to unnecessary and invasive treatments (non-maleficence); c) benefiting and not harming patients (beneficence); and d) reducing excessive costs (justice). In the example presented by Lysaght and colleagues for decision-making about AI-Assisted CDSS in End-of-life Care, the decisor will thus need to weigh up the potential benefits to patients in avoiding highly invasive interventions and more efficient use of ICU resources with the risks of some patients being denied intensive care due to poor predicted outcomes (Lysaght et al., 2019).

Second step: Getting the facts about the problem

At this level, the goal is to gather information from two areas: how the system works and how doctors use it. The first area is related to explainability and focuses on having a clear map of the data the application is using, and which data carries more weight in the outcome. The second area focuses on truly identifying how clinicians use the application and how it enhances their decision-making and communication with patients and their families.

Third step: Identifying the relevant and procedural values

This step requires a well-developed moral judgment that carefully balances the four bioethics principles. Professional integrity and public benefit serve as the foundation of the decision-making process. Certain values may take precedence over others depending on the patient’s health condition, context, and the decision-maker’s role. For example, as a hospital administrator, the principle of justice may take priority over patient autonomy. Conversely, as a doctor, the principle of non-maleficence may outweigh patient autonomy in cases of invasive treatments that cause excessive suffering with low survival prospects.

Fourth step: Considering the options and making an ethical decision.

In the example presented by Lysaght and colleagues, the options are to implement the AI-assisted CDSS into the ICU for prognostic scoring. Implementing this system benefits patients and their families by avoiding unnecessary, highly invasive interventions and setting realistic expectations for survival beyond the ICU.

The intention or aims when applying AI-assisted CDSS are crucial to its legitimacy. If the system were adopted solely or even primarily to save money, patients and their families would be justified in having concerns that the patient’s worth is being judged. However, if the primary aim were to avoid imposing significant burdens and harms on patients by providing non-beneficial treatments, the moral value of such efforts would be justified, as the patient would be at the centre of such considerations.

In cases where the AI CDSS resulted in a decision that conflicted with the health professionals, it would be imperative that the clinical team responsible for the patient understand the basis for the decision to ensure the technology provided maximum transparency. As the German ethics commission states: “crossing the line between decision-making assistance and adoption of decisions carries the risk of negligence or even loss of control” (Ethikkommission, 2021, p. 5)

In our view, this model proposed by Lysaght and colleagues is very interesting; however, we identify two weaknesses: first, it assumes a rationalist approach to moral decision-making in its four-step process, ignoring the intuitive and heuristic dimension; and second, it lacks a solid normative foundation for the process.

### ADC Moral Judgment Model Proposal

Following the work of Dubljević and colleagues (Dubljević & Racine, 2014, Dubljević, 2017; Dubljevic et al., 2018; Pflanzer et al., 2023), we propose the ADC model of moral judgment and examine the challenges of integrating ethical principles into AI CDSS. We concur with these authors in their assertion that: “models of human judgments and decision making, such as the agent-deed-consequence (ADC) model, will be crucial to inform the ethical guidance functions in AI teammates and to clarify how and why humans (dis)trust machines” (Pflanzer et al., 2023, p. 1).

As presented above, the ADC model of moral judgement considers that ethics is a complex realm of judgment and decision-making which encompasses decisions about Agents (e.g., is a particular person more virtuous?), Deeds (e.g., is lying justifiable?) and Consequences (e.g., could more people have been saved?) that must all be considered. This model is a powerful tool that can facilitate the development of ethical algorithms in which the weighted values of Agents, Deeds, and Consequences are moderated by a human moral agent, thus accommodating a variety of ethical frameworks. Though each is a valid way to evaluate ethical situations, these three theories – virtue ethics, deontology, and utilitarianism – are often applied differently to AI.

The integration process can be operationalised through three fundamental steps:

First step: Designing scenarios and modifying agents’ behaviour, situations, and outcomes. This step involves creating a variety of ethical decision-making scenarios in which an agent (A) must make a decision (D) that leads to specific consequences (C). These scenarios are then systematically adjusted by altering the moral characteristics of the three dimensions: A, D, and C. For example: a benevolent person (A+) lies (D-), resulting in harm to an innocent person (C-); or a delinquent (A-) tells the truth (D+), enabling another delinquent to go free (C-); or an honourable person (A+) keeps their promise (D+), causing a company to go bankrupt (C-); and so on. These scenarios are progressively modified and enriched with information to provide a wide variety of situations and increased realism.

Second step: Human experts evaluate these scenarios. The scenarios are analysed and evaluated by human experts. The experts assign scores to each dimension and provide justifications for each evaluation. A critical mass of experts would independently analyse multiple scenarios. Subsequently, meetings would be organised for experts to discuss and analyse various scenarios collectively. This is a central step, where deliberation is the key.

Third step: Training systems based on these cases and specific evaluations. The final step involves programming AI systems using this straightforward algorithm and training them using these evaluations. Human experts should then review the system’s results and recommendations to refine further and improve its performance.

## Discursive Ethics Framework for Embedding Ethical Principles within CDSS

Building on the two proposals discussed in the previous section regarding the incorporation of ethics into AI CDSS, we contend that effective AI integration requires a robust normative foundation. Providing a clear justification for specific procedures is essential to the legitimacy of the process and to cultivating trust among healthcare professionals. In this final section, we propose a discursive framework for embedding ethical principles within Clinical Decision Support Systems. This methodology is grounded in the discourse ethics of K.O. Apel (1973, 1988, 1997), Jürgen Habermas (1968, 1981, 1983, 2021), and the Spanish philosopher, Adela Cortina (1985, 1986, 1990).

### Discursive Ethics

Discourse ethics is rooted in the Kantian tradition of deontological, universalist, and formalist thinking. Its primary concern is to establish rational procedures for determining when a norm is valid. Unlike utilitarian approaches that focus on means–ends rationality, discourse ethics emphasises communicative rationality and the rationality of ends, moving beyond individualistic ethics toward intersubjective dialogue.

At its core, discourse ethics holds that moral norms can claim validity only insofar as they can be justified through rational dialogue among those affected by them. The two main authors of this theory are Karl-Otto Apel and Jürgen Habermas.

The philosophical foundation of this approach lies in the linguistic pragmatic-transcendental turn (Apel, 1973), which argues that anyone who engages in argumentation implicitly accepts certain normative conditions that make rational discourse possible. These conditions rest on the double a priori of the communication community: first, the argumentative a priori, which presupposes mutual recognition of all participants as equal and mature interlocutors; and second, the experiential a priori, which acknowledges that real-life communication is essential for personal and cultural development.

K. O. Apel distinguishes two main components of discourse ethics. Part A provides the abstract foundation, including the transcendental basis for norm justification and situational norms for practical discourse. It delegates concrete norm-setting to those affected, ensuring both universality and responsibility. Part B addresses historical applications, emphasising responsibility for preserving natural and cultural resources and for adapting ethical principles to real-world conditions. At this level, discourse ethics acknowledges that ideal conditions rarely exist in practice and therefore must be complemented by strategic rationality to safeguard survival and systemic stability.

In a similar vein, though grounded in a different philosophical framework, Habermas’s theory of communicative action aims at achieving mutual understanding. Habermas introduced the concept of an ideal speech situation structured by three core requirements: (a) all potential participants must have equal opportunities to take part in discourse; (b) participants must be free to question any claim, introduce new assertions into the discussion, and express their own attitudes, needs, or preferences; and (c) no participant should be prevented—through internal or external, overt or covert coercion—from exercising these rights.

Under these conditions, a dialogically adequate process includes all relevant stakeholders, grants participants equal standing, and evaluates competing arguments solely on their merits, free from coercive influence. Habermas maintains that these conditions are not merely conventional but constitute unavoidable presuppositions of rational argumentation. Accordingly, a participant who engages in argumentation while rejecting these presuppositions either acts strategically (through deception) or incurs a performative contradiction.

Habermas initially conceived the ideal speech situation as a regulative idea for guiding and evaluating real-world discourse (Habermas, 1981). In later work, however, he reformulated this approach in terms of two complementary principles: the discourse principle and the universalisation principle. The discourse principle (D)—“Only those norms can claim to be valid that meet (or could meet) with the approval of all affected in their capacity as participants in a practical discourse” (Habermas, 1992, p. 66)—articulates the procedural conditions of justification, whereas the universalization principle (U)—“A norm is valid when the foreseeable consequences and side effects of its general observance for the interests and value-orientations of each individual could be jointly accepted by all concerned without coercion” (Habermas, 1983, p. 42)—provides the substantive moral test.

Within this framework, discourse ethics emphasises the rationality of consensus-formation processes. It prioritises procedural rationality and the “force of the better argument” over power asymmetries or strategic interests, without presupposing substantive moral commitments. Genuine discourse, therefore, requires institutional and communicative conditions that secure equality among participants, shared responsibility in deliberation, and a commitment to resolving disagreements through reasoned agreement.

Adela Cortina (Cortina, 1985, 1986, 1990, 1993, 1996, 1997, 2000, 2010), drawing on the tradition of discourse ethics, proposes civic ethics as an attempt to apply its normative principles to institutions and social practices. She understands this application not as a deductive or inductive process, but rather as a form of critical hermeneutics.

This hermeneutical approach pays particular attention to decision-making procedures in concrete contexts and, in this sense, represents a higher level of practical specification than the conditions of application proposed by Karl-Otto Apel and Jürgen Habermas. The process of hermeneutical integration unfolds in three steps: (a) identifying the goal or internal good (telos) of each institution or social practice; (b) developing as complete and detailed an understanding as possible of the specific context in which the principles are to be applied; and (c) proposing the specific values and principles that emerge from the configuration of the dialogical ethical principle within that particular activity or institution. Through this approach, Cortina develops a proposal that takes into account the deontological moment of normative grounding while simultaneously incorporating an awareness of the ends of human activity, the complexity of the real world, and the responsibility for the future.

This hermeneutical application assumes the idea of the subject as a valid interlocutor, a notion common to all institutions and professional and social practices. According to this view, those affected are ultimately the ones legitimately entitled to present their interests and to ensure that universalisable interests are taken into account.

Institutionalising discourse ethics is a critical challenge that, for decades, has been pursued in different fields, such as Business Ethics (Cortina, 1994; Lozano, 2003; Ulrich, 1997), Bioethics (Siurana, 2010), Information Systems (Mingers & Walsham, 2010), and IA (Sinha & Derichs, 2025). (Xivuri & Twinomurinzi, 2022) proposed Habermas’ theory of communicative action as an ideal mechanism to review AI algorithms to improve their fairness before and after implementation. William Rehg analyses two controversial cyberpractices based on AI systems (violent games and NSA surveillance) using Habermasian universalisation and dialogical robustness principles (Rehg, 2015).

Our opinion is that the discursive approach has essential traits that make it especially suited to the challenges of integrating ethics into AI CDSS and that it overcomes three main ideas that prevail in the science-society relationship: paternalism, self-interest, and individualism.

No paternalism. The dialogue between those affected is a legitimate process for deciding what to do in concrete situations. This process dismisses the idea of experts in a superior epistemological position who make unilateral calculations based on technical knowledge and causal logic, without considering the opinions of those affected by the decision. Xivuri and Twinomurinzi (2022) and Rehg (2015) agree that individuals affected by AI algorithms or by decisions made by AI algorithms should be involved in the planning and development stages of AI algorithms. All stakeholders, developers, and society directly affected by AI should be aware of the discursive requirements set out by and apply them during the development and implementation of AI.

No self-interest. The dialogical approach aims to achieve consensus among those affected by norms and decisions. This approach transcends agreements based on the interests of those with the power to participate in negotiations, which also supposes that universal principles, values and common goals are prioritised over particular and private interests.

No individualism. From a philosophical perspective, this dialogic responsibility assumes intersubjectivity and mutual acknowledgement as a basis for decision-making and sees rationality as the capacity to deal not just with objects but also with people, and to discuss not just means but also ends.

### Practical Implications of the Discursive Approach for Integration Processes

This model assumes the principle that humans should supervise an AI system. It offers the following advantages.

Human Factor Involvement. The system incorporates human oversight throughout its design, application, and evaluation, ensuring that ethical standards and societal norms are embedded from the start. As Pardoux and Kerasidou have recently highlighted, the idea of “Compliance by design” (CbD) is especially suited to AI clinical decision-making in contexts with high risk and low tolerance to non-compliance (Pardoux & Kerasidou, 2025). It avoids becoming an opaque “black box” by integrating human values and clinical guidelines at every level. For instance, a system designed for medical triage in emergencies would involve healthcare professionals who understand the ethical implications of prioritising patients, ensuring the system’s decisions align with real-world healthcare ethics and good clinical practices.

Ethical Expertise. Human input comes from individuals trained in ethical reasoning and moral philosophy. They focus on reflective judgment, not just the technical application of rules or descriptive analytics. This ensures the system incorporates nuanced ethical considerations. For example, in an AI system used for hiring, ethical experts could ensure that the algorithms avoid discriminatory practices and reflect a rigorous understanding of fairness, diversity, and inclusion.

Argument-Based Foundation. The system’s decision-making relies on structured, argument-based reasoning rather than arbitrary rules. Each decision can be supported by a chain of logical justifications and solid philosophical arguments, making the model robust and defensible. For instance, a legal decision-support system that recommends case outcomes could document its reasoning process based on legal precedents and ethical arguments, enabling lawyers and judges to scrutinise its conclusions.

Simplicity and Clarity. The system is straightforward, allowing each decision to be traced back to its origins. This traceability makes the model easier to audit, debug, and improve. For instance, in financial AI systems used for loan approvals, the model’s simplicity and transparency could allow applicants to see how their creditworthiness is assessed, reducing distrust in the system.

User Accessibility. Transparency is prioritised to enable end-users to understand how the system operates. This promotes trust and empowers users to challenge decisions if they believe errors or biases exist. For example, a recommendation system in education used to guide students’ career paths could present its logic in a way that students and educators can comprehend, helping them make informed decisions collaboratively.

Moral Alignment Without Bias. The system’s logic mirrors human moral reasoning, making its decisions relatable and acceptable to society. However, it is designed to exclude biases such as favouritism or emotional reasoning, leading to more impartial outcomes. For instance, autonomous vehicles could use such a system to make life-and-death decisions during accidents, applying ethical principles like utilitarianism (maximising overall safety) while avoiding subjective biases.

We are convinced that a discursive ethical framework provides a compelling normative foundation for the responsible integration of AI into Clinical Decision Support Systems. By prioritising inclusive dialogue, shared responsibility, and the rational justification of norms, this approach strengthens both the legitimacy and trustworthiness of AI-supported clinical decisions. As AI systems increasingly shape high-stakes healthcare contexts, institutionalising such a discursive model offers a practical and philosophically robust pathway to ensure that technological innovation remains ethically grounded and socially responsive.

## Conclusion

In the rapidly evolving landscape of Artificial Intelligence, the integration of ethical principles has transitioned from a theoretical preference to a pressing normative and existential necessity. This paper hypothesises that the development of Clinical Decision Support Systems (CDSS) cannot be evaluated solely through the lenses of algorithmic efficiency, predictive accuracy, or computational speed. Instead, these systems must be legitimised by their capacity to enhance human agency, safeguard dignity, and promote social flourishing within a complex healthcare environment.

The application of discursive ethics—rooted in the work of Apel, Habermas, and Cortina—provides a robust framework to overcome the limitations of purely instrumental or functionalist approaches. By prioritising communicative rationality, the proposed integration ensures that AI does not operate as an opaque “black box” imposing decisions, but rather as a transparent mediator that facilitates critical judgment and mutual understanding. The legitimacy of AI in clinical settings is intrinsically linked to its “accountability to the affected”; systems must be designed to be explainable and subservient to the ethical deliberations of human actors. Furthermore, this study concludes that the transition away from traditional medical paternalism must not lead to a new form of “algorithmic authoritarianism.” On the contrary, ethical integration into AI architecture must reinforce the clinician’s professional responsibility and the patient’s informed autonomy.

Ultimately, achieving a truly ethical AI requires a continuous, transdisciplinary effort. It is not enough to implement technical protocols or “ethics by design” as a mere checklist. It demands a profound dialogue between moral philosophy, data engineering, and public policy. By designing systems that augment human capabilities without diminishing moral responsibility, we can ensure that technological innovation catalyses a more just, equitable, and sustainable society.

## Data Availability

Not applicable.
